# Crown Gall Induced by a Natural Isolate of *Brucella* (*Ochrobactrum*) *pseudogrignonense* Containing a Tumor-Inducing Plasmid

**DOI:** 10.3390/microorganisms13010102

**Published:** 2025-01-07

**Authors:** Marjolein J. G. Hooykaas, Paul J. J. Hooykaas

**Affiliations:** Institute Biology Leiden (IBL), Leiden University, 2333 BE Leiden, The Netherlands; marjolein.hooykaas@wur.nl

**Keywords:** crown gall, *Agrobacterium*, *Brucella*, *Ochrobactrum*, Ti plasmid, succinamopine type Ti

## Abstract

Crown gall disease in plants is caused by “Agrobacteria”, bacteria belonging to the *Rhizobiaceae* family, which carry a tumor-inducing (Ti) plasmid. Unexpectedly, we found evidence that a natural isolate from a rose crown gall, called NBC51/LBA8980, was a bacterium that did not belong to the *Rhizobiaceae* family. Whole-genome sequencing revealed that this bacterium contained three large DNA circles with rRNA and tRNA genes, representing one chromosome and two chromids, respectively, and two megaplasmids, including a Ti plasmid. Average nucleotide identity (ANIb, ANIm) and genome-to-genome distance (GGDC) values above the thresholds of 96% and 70%, respectively, showed that NBC51/LBA8980 belonged to the species *Brucella* (*Ochrobactrum) pseudogrignonense.* Its Ti plasmid was almost identical to certain succinamopine Ti plasmids previously identified in *Agrobacterium* strains, suggesting that this Ti plasmid may have been recently acquired by NBC51/LBA8980 in the tumor environment.

## 1. Introduction

Crown gall, an overgrowth that can occur on many plant species, is caused by the bacterium *Agrobacterium tumefaciens*. Because of its importance as a phytopathogen and for its use in plant biotechnology, the bacterium has been intensely studied over the last 50 years. The molecular mechanism by which the bacterium induces crown gall has been elucidated over the years. The results are briefly summarized in the following section, with references to reviews for further reading [1,2,3,4,5,6,7,8,9]. Crown galls are characterized by the presence of unusual compounds called opines, which are not found in normal plant cells or in *Agrobacterium* [1]. Remarkably, the bacterium was found to genetically modify plant cells at wound sites, turning them into tumor cells [2]. The bacterial DNA segment introduced into plant cells, called T-DNA, contains a number of genes that are expressed in plant cells, including various genes which encode enzymes for the production of the plant growth regulators indole acetic acid and isopentenyl-adenine. The T-DNA was also found to contain genes for the enzymes involved in the biosynthesis of opines. The T-DNA is derived from a large plasmid in the bacterium, the tumor-inducing (Ti) plasmid [2,3,4,5,6]. This plasmid also encodes some 20–30 virulence proteins involved in T-DNA transfer and transformation [4,5,6,7,8,9]. The T-DNA transfer system is evolutionarily derived from the bacterial conjugation system and delivers not only T-DNA (in single-stranded form) into the host cells, but also five virulence (effector) proteins [9]. In addition to a T-region and a virulence region, the Ti plasmid contains a *repABC* replicator, genes for conjugative transfer (between bacteria), and genes for the uptake and catabolism of the opines produced in the crown gall tumors [4,5,6,7,8,9]. A variable number of genes with unknown functions may also be present. Initially, two different classes of *Agrobacterium* strains were distinguished, octopine and nopaline strains. While both types induced crown gall tumors, octopine strains induced octopine-containing tumors and had the ability to degrade octopine, whereas nopaline strains could only degrade nopaline produced in the tumors they induced [1]. Over time, other opine types have been discovered. DNA sequencing has shown that Ti plasmids can be classified based on the opine synthases on their T-DNA [10]. Since the Ti plasmid determines the phytopathogenic properties of the bacterium, it was recognized that different types of tumor-inducing bacteria could actually harbor the Ti plasmid. These were initially classified as biotypes 1–3 on the basis of their growth characteristics [11]. These “agrobacteria” all belong to the family *Rhizobiaceae*, but according to current taxonomy, biotype 1 strains belong to different species within the genus *Agrobacterium*, biotype 2 strains to species within the genus *Rhizobium*, and biotype 3 to multiple species in the genus *Allorhizobium* [12].

## 2. Materials and Methods

### 2.1. Bacterial Culture, DNA Isolation, and Virulence Test

Strain NBC51/LBA8980 was obtained from NAKtuinbouw, Roelofarendsveen, the Netherlands (M. Ebskamp, E. Meekes). The bacterium was grown on TY medium (Difco tryptone 5 g/L, Difco yeast extract 3 g/L, CaCl_2_.6H_2_O 1.3 g/L) and tested for virulence by puncturing the plant stems with a sterile wooden toothpick that had been dipped in a colony of the bacterium. Genomic DNA was isolated using QIAGEN genomic-tip gravity-flow columns (QIAGEN Benelux, Venlo, The Netherlands). 

### 2.2. Sequencing Methods 

An initial genome assembly was obtained after Illumina sequencing at Baseclear (W. Pirovano, Leiden, The Netherlands), where Illumina paired-end sequence reads were generated using an Illumina HiSeq2500 system, providing 582,823,698 total bases and 4,631,674 quality filtered paired-end reads. The complete genomic sequence was obtained, but was distributed over 47 scaffolds. In order to obtain a high-quality genomic sequence, additional long-read Nanopore sequencing was performed in-house using Oxford Nanopore Technologies platforms. The Oxford Nanopore sequencing library was generated from 200 ng DNA using the SQK-RBK004 Rapid Barcoding Kit (Oxford Nanopore Technologies, Oxford, UK). The library was pooled with another library, followed by in-house sequencing on a MinION flow cell (version R9.4.1).

### 2.3. Data Processing Methods

After basecalling with Albacore (version 2.3.4), the MinION reads were demultiplexed (with Epi2me). The total yield for NBC51/LBA8980 was 312,509 reads totaling 1.15 × 10^9^ bp, with a mean read length of 3682 bp. The nanopore reads were end-trimmed and filtered for average quality (>Q10) and length (>5000 bp) with NanoFilt (64-fold coverage after filtering). A hybrid assembly (combining the filtered Nanopore and Illumina reads) was obtained using Unicycler version 0.4.7, which resulted in five contigs representing the chromosomes and the plasmids of NBC51/LBA8980. The assembly was annotated using the NCBI Prokaryotic Genome Annotation Pipeline (PGAP, [13], see NCBI website), and using the RAST server [14], as shown in Table S1. Protein assignments to the Clusters of Orthologous Groups (COGs) functional categories were obtained with eggNOG-mapper v2 [15] using an online server (http://eggnog-mapper.embl.de, accessed on 21 November 2024). Average nucleotide identity (ANI) values were determined on the JSpeciesWS online server (https://www.ribocon.com/jspeciesws.html, accessed on 28 November 2024) using BLASTn (ANIb) and MUMmer (ANIm) against the GenomesDB reference database of 65,774 genomes, including those of 24,708 types of strains [16]. Digital DDH (dDDH) values of the genome-to-genome distances (GGDC, [17,18]) were obtained by a pairwise comparison of NBC51/LBA8980 with other genomes using GGDC 2.1 (identities/HSP length) online (https://ggdc.dsmz.de, accessed on 30 November 2024).

### 2.4. Data Availability

The complete genome sequence of strain NBC51/LBA8980 has been deposited in GenBank (BioSample SAMN45108879, Accession: PRJNA1192426; CP175669-CP175673).

## 3. Results

### 3.1. Crown Gall-Inducing Strain NBC51/LBA8980

**Figure 1:**
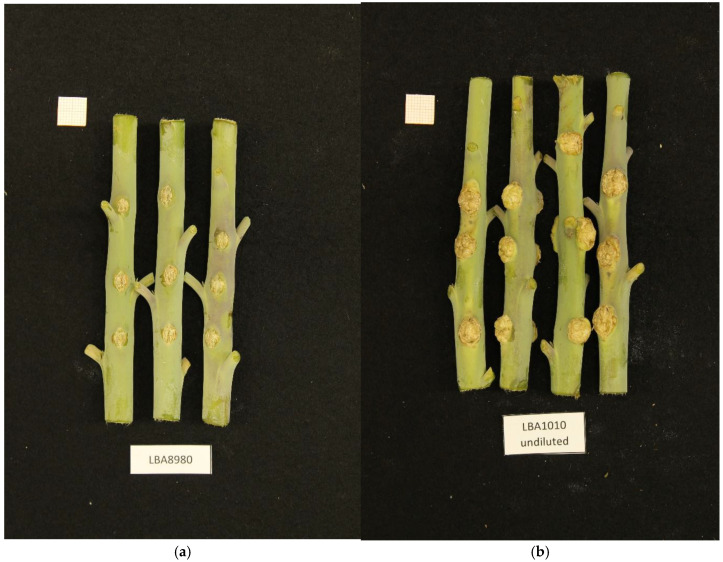
(**a**). Small crown gall tumors induced in *Nicotiana glauca* by NBC51/LBA8980. (**b**). For comparison crown gall tumors induced by a common laboratory strain LBA1010 (C58 with pTiB6).

A number of bacterial strains were isolated from crown gall tumors in the Netherlands and characterized for growth characteristics and virulence by NAKtuinbouw (Roelofarendsveen, The Netherlands). A preliminary analysis of the genomic DNA by Illumina short-read sequencing revealed that one of these strains did not belong to any of the *Agrobacterium* or *Rhizobium* species known to cause crown gall. This strain, called NBC51 (LBA8980 in our collection), was isolated from a rose gall in 1997, and was able to induce crown gall on rose and tomato (Epskamp and Meekes, personal communication). We tested this isolate for crown gall formation on *Kalanchoe tubiflora* and *Nicotiana glauca* and found that the strain could indeed induce crown gall on these plants, although the tumors were small compared to those induced by commonly used laboratory *Agrobacterium* strains (Figure 1).

### 3.2. Assembly of a High Quality, Complete NBC51/LBA8980 Genome

To characterize its genomic DNA in detail, we isolated high-molecular-weight genomic DNA for long-read Nanopore sequencing and combined the reads with those previously obtained by Illumina short-read sequencing (using Illumina reads a preliminary genome sequence of 47 scaffolds was assembled earlier) for hybrid genome assembly using Unicycler. We obtained a complete, high-quality genome sequence. The NBC51/LBA8980 genome has a total size of 5,328,147 bp with 53.6% GC content, for which 5281 protein-coding genes are predicted, as well as sixty tRNA genes and five copies each of the 5S rRNA, 16S rRNA, and 23S rRNA genes. The chromosome, the largest DNA circle characterized by the presence of a *dnaA* replication initiation gene, has a size of 2,409,785 bp; it contains two copies of the rRNA operons and forty of the tRNA genes. In addition to the chromosome, four other DNA circles were identified with sizes of 1,731,835 bp, 876,982 bp, 180,077 bp, and 129,468 bp, respectively. These large DNA circles do not encode a DnaA replication initiation protein, but rely on a *repABC* system for replication, as do most plasmids in the bacterial order *Hyphomicrobiales,* to which the *Rhizobiaceae* belong. The two largest of these plasmids, like the chromosome, contain rRNA and tRNA genes, two copies of each of the rRNA genes and 17 tRNA genes in the largest plasmid, and one copy of the rRNA genes and three tRNA genes in the 876 kbp plasmid. These large plasmids appear to be developing into secondary chromosomes, chromids [19], and will be referred to hereafter as chromosome 2 and chromosome 3. They have a GC content of 53.1% (chromosome 2) and 54.7% (chromosome 3), which is slightly different from that of chromosome 1 (53.5% GC). The remaining DNA circles, plasmid4, which is the Ti plasmid pTi51 (see below for details), and the smallest circle, plasmid5 (now called plasmid pOp51), have a GC content that is more different from that of chromosome 1 with 56.1% GC for the Ti plasmid and 51.2% GC for pOp51.

### 3.3. NBC51/LBA8980 Belongs to the Species Brucella (Ochrobactrum) pseudogrignonense

The availability of a high-quality genome sequence of strain NBC51/LBA8980 allowed the use of new genomics tools to classify the strain. We used the online server JSpeciesWS (https://jspecies.ribohost.com/jspeciesws/, accessed on 28 November 2024) to compare the genome of NBC51/LBA8980 with the reference database GenomesDB, which contains more than 60,000 genomes. The Tetra Correlation Search function (TCS) revealed that NBC51/LBA8980 was most closely related to *Ochrobactrum* sp. CDB2 and *Brucella pseudogrignonense* strains, including type strain CCUG 30717^T^ (Table S1). This was confirmed by determining the average nucleotide identity (ANI) using BLASTn (ANIb) and MUMmer (ANIm) to perform alignments against the same reference database on the JSpeciesWS online server (Table 1). The high nucleotide identity of 97–98% found for these two bacteria (over approximately 87% of the genome) is above the recommended ANI limit for species discrimination of 95–96% [16,20]. In similar analyses, other *Brucella* species were found to share a much lower genomic identity over much smaller portions of the genome. Strain NBC51/LBA8980 thus appears to belong to the species *Brucella (Ochrobactrum) pseudogrignonense,* until recently named *Ochrobactrum pseudogrignonense.* We then used the Genome BLAST Distance Phylogeny (GBDP)-based digital DDH (dDDH) method online (https://ggdc.dsmz.de, accessed on 30 November 2024) to validate this result. This revealed a genome-to-genome distance (GGDC) value of over 80% between NBC51/LBA8980 and CCUG 30717^T^, the type strain of *Brucella pseudogrignonense,* which is well above the classical cut-off point of 70% DDH for species delimitation [17,18].

### 3.4. Functions Encoded by the Chromosomes and the Plasmids

The genome was annotated with the RAST (Rapid Annotations using Subsystems Technology) server [14] as shown in Table S2, as well as with the NCBI Prokaryotic Genome Annotation Pipeline (PGAP [13]) at the web site. Chromosome 1 contains most of the rRNA and tRNA gene clusters, as well as the majority of the genes required for genome maintenance and replication for ribosomal proteins and translation. A complete set of genes for a gene transfer agent (GTA) are present in chromosome 1. Such genes were first identified in *Rhodobacter capsulatus*, but have since been identified in other alphaproteobacteria. They encode phage-like particles that facilitate gene transfer [21]. Chromosome 2, one of the chromids, also contains some rDNA and tRNA genes, as well as some genes involved in genome maintenance and repair, translation, and transcription, but also several metabolic genes and several genes encoding siderophores. Chromosome 3, the other chromid, has the fewest rRNA and tRNA genes of the three and is the most plasmid-like, carrying a full set of conjugation genes. It also contains some DNA maintenance and repair genes, including an *imuABC* cluster for SOS mutagenesis as well as Ku and LigC genes for non-homologous end-joining, and genes for metabolic and transport functions, again including siderophore gene clusters. Plasmid4 is the Ti plasmid discussed below. Plasmid5 is probably a conjugative plasmid as it contains a full set of conjugative genes. It contains many genes of unknown function and has very little homology to any plasmid or chromosomal DNA in the NCBI database.

### 3.5. Ti Plasmid pTi151

**Figure 2:**
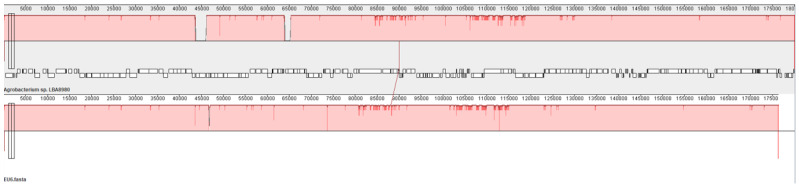
Pairwise alignment of pTi51 (top LBA8980) and pTiEU6 (bottom EU6) by progressive MAUVE [25]. The large gap in the top (pTi51) represents the transposable element inserted in the *trbI* gene.

Plasmid4 is the Ti plasmid containing the genes essential for crown gall formation. Nucleotide Blast (NCBI) showed that pTi51, the name we propose for this plasmid, is almost identical to plasmid pTi186 identified in an *Agrobacterium* strain isolated from walnut [22] and also to plasmid pTi1D132 from an *Agrobacterium* strain isolated from a cherry gall [23]. Apart from a few single-nucleotide polymorphisms (3 snp’s with pTi186; 12 snp’s with pTi132), the only difference found was the insertion of a transposable element in the *trbI* gene in pTi151, which probably rendered this plasmid non-conjugative. These three plasmids are also very similar (99.7% nucleotide identity over almost the entire length of the plasmid) to plasmid pTiEU6, which has been analyzed in detail in [24]. Plasmid pTiEU6 is a succinamopine Ti plasmid with the *susD* gene in the T-DNA region for the production of D,L-succinamopine in plant tumors [24]. This *susD* gene and the genes for succinamopine catabolism are conserved in pTi51. Also, the virulence genes and the T-region are fully conserved. We can therefore conclude that pTi51 is a succinamopine Ti plasmid. A pairwise alignment of pTi51 and pTiEU6 is shown in Figure 2.

## 4. Discussion

Crown gall bacteria were originally divided into three biotypes [11]. These biotypes represent the genera *Agrobacterium*, *Rhizobium,* and *Allorhizobium,* all of which belong to the bacterial family *Rhizobiaceae* [12]. This is also the case for the *Neorhizobium* sp. strain carrying a Ti plasmid recently isolated from a crown gall in Taiwan [26]. However, we now add the genus *Ochrobactrum/Brucella* to the list of bacteria that can naturally carry a Ti plasmid and thus become pathogenic to plants. As the genus *Ochrobactrum/Brucella* does not belong to the bacterial family *Rhizobiaceae*, but to the bacterial family of the *Bartonellaceae* [27], strain NBC51/LBA8980 is the first natural isolate outside the *Rhizobiaceae* capable of inducing crown gall. This is not entirely unexpected, as laboratory experiments in the past have shown that transferring the Ti plasmid to other host bacteria can render them virulent. Initially, such experiments were restricted to different strains of the closely related bacterium *Rhizobium leguminosarum* [28], but later, more distant bacteria were also used as recipients, including *Phyllobacterium myrsinacearum* [29,30]. The latter host, which, like *Ochrobactrum,* becomes tumorigenic after receiving the Ti plasmid, does not belong to the *Rhizobiaceae* family. Transferring the Ti plasmid to bacteria outside the Alphaproteobacteria such as *Escherichia coli* could also be achieved in the form of a cointegrate with a broad host range R plasmid. However, such bacteria did not become plant pathogenic [31]. It may be relevant to mention here that the Ri plasmid, which is carried by hairy root-inducing *Agrobacterium* and *Rhizobium* strains, has previously been found in *Ochrobactrum* strains in greenhouse tomatoes and cucumbers affected by root mat disease [32]. Interestingly, it was also recently reported that the bacterium responsible for a tumor disease on the mushroom *Flammulina velutipes* was classified as *Ochrobactrum pseudogrignonense* [33]. It will be very interesting to identify the genes responsible for infection from this bacterium, as well as their origin.

Only small tumors are induced by strain NBC51/LBA8980. This may be due to the absence of certain chromosomal virulence factors. However, homologs of the known chromosomal virulence genes *chvAB*, *chvGI*, and *acvB* were identified in the genome, but whether these are functional remains to be tested. The formation of smaller tumors in novel bacterial backgrounds was previously observed when the Ti plasmid was introduced into novel bacterial hosts such as *Rhizobium leguminosarum* and *Phyllobacterium myrsinacearum* in the laboratory [29,30]. A *Sinorhizobium meliloti* strain receiving the Ti plasmid was even completely deficient in inducing crown gall in plants [34]. It may well be that the reduction or absence of tumorigenicity in these new host bacteria is due to a lack of co-evolution between the Ti plasmid and the host genome. In nature, new tumorigenic species may eventually evolve through adaptive changes in the Ti plasmid and the genome. Otherwise, the Ti plasmid may be lost from such new hosts or, if this is difficult, for example, due to the presence of toxin–antitoxin systems on the Ti plasmid, lead to degeneration of the Ti plasmid. The inactivation of the conjugation system by a transposable element in pTi151 may be the first step in this direction in NBC51/LBA8980. The presence of a Ti plasmid that had lost most of its T-region in a *Rhizobium etli* strain isolated from soil [6,35] also points in this direction. Conversely, specific Ti plasmids seem to have evolved in *Rhizobium tumorigenes* and *R. rhododendri* that allow tumor formation on blueberry and rhododendron [36], and this has also been described for Ti plasmids in *Allorhizobium vitis* that allow tumor formation on *Vitis vinifera* [37].

Recently, disarmed Ti plasmids have been introduced into several different host bacteria and tested for their ability to transfer selectable genes present on the T-region of a binary vector into plants [38,39]. Several of these species, including *Mesorhizobium loti* [38] and *Ensifer adhaerans* [39], were found to be capable of transferring genes into plants. Remarkably, *S. meliloti* was one of the species able to transfer genes into plants [38]. Whether this positive result was due to the use of a different strain of *S. meliloti* than that used to transfer the natural Ti plasmid, or to the use of a different assay system to detect transfer (growth of transformed plant cells on selective medium instead of crown gall tumor formation) requires further study. Interestingly, one of the bacteria that was selected for use as a plant vector in an even more recent experiment was a bacterium that was called *Ochrobactrum haywardense* H1 [40]. It is therefore possible that the *Ochrobactrum/Brucella* isolate described here may have similar useful properties to a plant gene vector. To test this, the Ti plasmid present in strain LBA8980 needs to be disarmed or replaced by one of the existing disarmed helper Ti plasmids.

For the practical application of such a disarmed strain, it would be important to verify that the strain lacks human/animal pathogenicity factors that are prevalent in pathogenic *Brucella* strains. This is likely to be the case as pairwise comparisons of hundreds of genomes revealed a clear separation of the pathogenic *Brucella* strains from the environmental strains previously referred to as *Ochrobactrum* strains [41]. This is also consistent with genomic studies showing that major genomic rearrangements have occurred in the *Brucella* genome over time, with dozens of genes present in chromosome 1 of *Ochrobactrum* moving to chromosome 2 [42]. At the same time, certain metabolic genes were inactivated by mutation, resulting in the evolution of zoonotic *Brucella* species towards the selective use of the pentose phosphate pathway for glucose catabolism [43]. All of this adds weight to the criticism of the decision to include the environmental *Ochrobactrum* strains in the genus *Brucella,* which traditionally contains zoonotic species [44,45]. Strain NBC51/LBA8980 belongs to the group of environmental strains (*Ochrobactrum*) because it still has the above-mentioned genes on chromosome 1 and lacks the mutations that restrict glucose catabolism through the pentose phosphate pathway. Nevertheless, *Ochrobactrum* has also been found as an opportunistic human pathogen in hospitals [46]. In this respect it is no different from *Agrobacterium* [47].

## Figures and Tables

**Table 1:** Pairwise comparison of the NBC51/LBA8980 genome with the most closely related *Brucella/Ochrobactrum* strains by ANIm using the JSpeciesWS online server [16].

	ANIm [%]	Aligned [%]	Aligned [bp]	Total [bp]
*Ochrobactrum* sp. CDB2	98.00	87.91	4,684,215	5,328,147
*Brucella pseudogrignonensis* CCUG 30717 [T]	97.87	88.24	4,701,446	5,328,147
*Brucella pseudogrignonensis* K8	97.76	84.63	4,509,247	5,328,147
*Brucella pituitosa* DSM 22207 [T]	86.57	55.14	2,937,694	5,328,147
*Brucella anthropi* ATCC 49188 [T]	84.89	23.18	1,235,081	5,328,147
*Brucella intermedia* LMG 3301 [T]	84.68	22.27	1,186,669	5,328,147

## Data Availability

DNA sequencing data are available at GenBank.

## Supplementary Materials

The following supporting information can be downloaded at: https://www.mdpi.com/article/10.3390/microorganisms13010102/s1, Table S1: List of genomes most closely related to strain NBC51/LBA8980 found in the GenomesDB database; Table S2: RAST annotation of the *Brucella (Ochrobactrum) pseudogrignonense* NBC51/LBA8980 genome.
