# Supplementary figures and images for: Crown Gall Induced by a Natural Isolate of Brucella (Ochrobactrum) pseudogrignonense Containing a Tumor-Inducing Plasmid
Source: Microorganisms. 2025 Jan 7;13(1):102. doi: 10.3390/microorganisms13010102 (PMC11767727; doi:10.3390/microorganisms13010102)

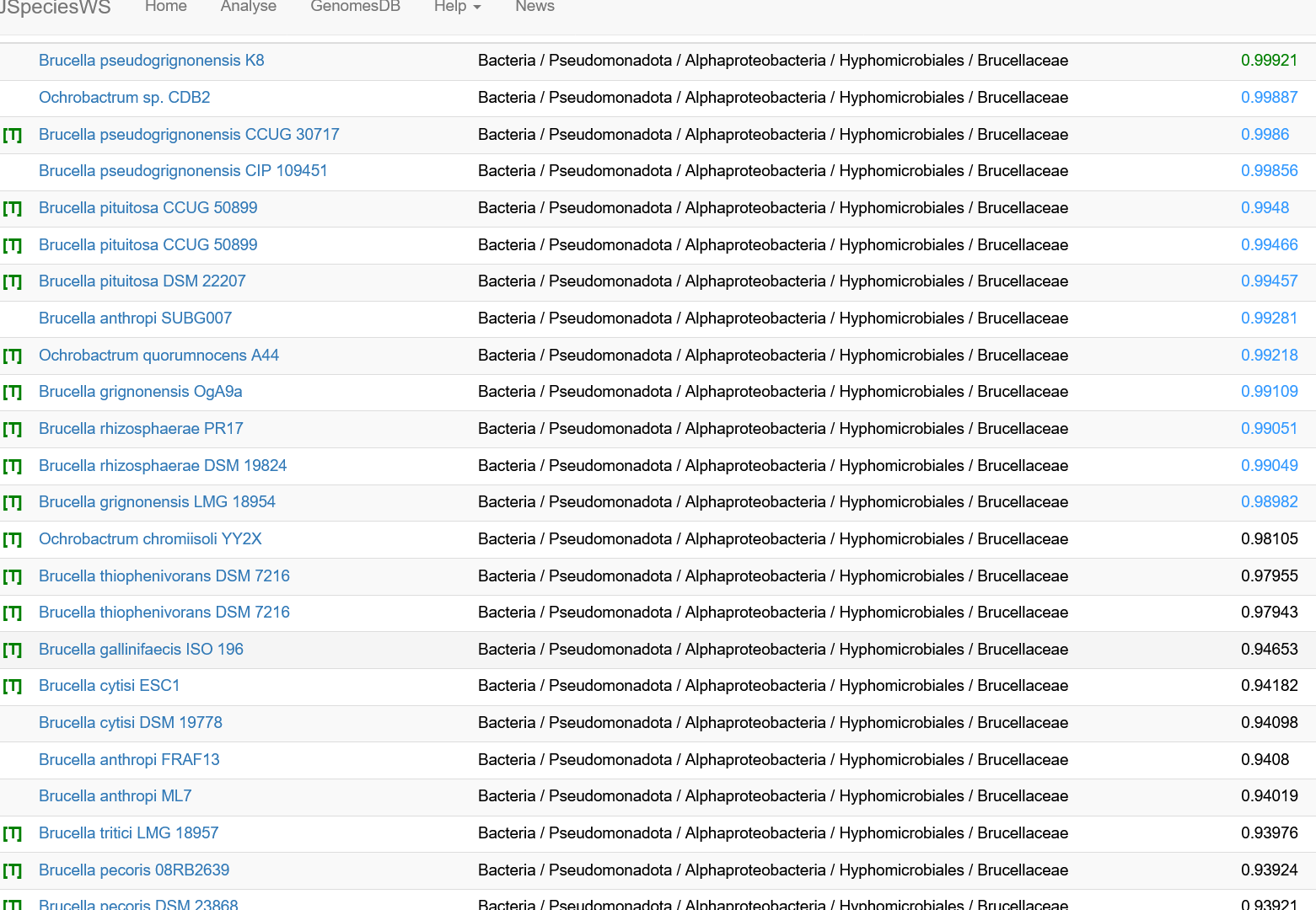

Supplement: Supplementary file 1 [file microorganisms-13-00102-s001.zip › Table S1. Genomes closely related to LBA8980 from JSpeciesWS .png]
